# Psychosocial factors at work and teachers’ illness: a systematic review

**DOI:** 10.47626/1679-4435-2022-1014

**Published:** 2023-11-24

**Authors:** Nayara Ribeiro Gomes, Caroline Castro de-Assis-Santos, Bárbara Antunes Rezende, Adriane Mesquita de-Medeiros

**Affiliations:** 1 Pós-Graduação em Ciências Fonoaudiológicas, Faculdade de Medicina, Universidade Federal de Minas Gerais (UFMG), Belo Horizonte, MG, Brazil; 2 Pós-Graduação em Saúde Pública, Faculdade de Medicina, UFMG, Belo Horizonte, MG, Brazil

**Keywords:** schoolteachers, occupational diseases, psychosocial impact, working conditions, occupational health, professores escolares, doenças profissionais, impacto psicossocial, condições de trabalho, saúde ocupacional

## Abstract

The aim of the present study is to analyze scientific evidence about associations between psychosocial factors at work and teachers’ illness. A systematic literature review based on the PRISMA statement was conducted. Biblioteca Virtual em Saúde, Medical Literature Analysis and Retrievel System Online, Cumulative Index of Nursing and Allied Health Literature, Scopus, Web of Science, PsycINFO, and Excerpta Medica Database databases were searched. Articles in Portuguese, English and Spanish, published in the past 11 years, were of interest. In total, 861 articles were identified, but only 15 of them met all the eligibility criteria and were included in the review. Eleven articles (73.3%) used validated instruments to assess psychosocial factors, and the Job Content Questionnaire was the most cited one. Low social support, heavy workload, high job demands, and low job control were the most commonly investigated factors and showed statistically significant associations with teachers’ illness.

## Abstract (pt)

O objetivo do presente estudo é analisar as evidências científicas sobre as associações entre os fatores psicossociais do trabalho e o adoecimento de professores. Desenvolveu-se uma revisão sistemática da literatura de acordo com a declaração PRISMA, nos bancos de dados Portal Biblioteca Virtual em Saúde, Medical Literature Analysis and Retrieval System Online, Cumulative Index of Nursing and Allied Health Literature, Scopus, Web of Science, PsycINFO e Excerpta Medica Database, em idioma português, inglês e espanhol e publicados nos últimos 10 anos. Foram identificados 861 estudos, dos quais 15 preencheram os critérios de seleção e foram incluídos na revisão. Onze estudos (73,3%) utilizaram instrumentos validados para avaliação dos fatores psicossociais, sendo o Job Content Questionnaire o mais citado. Os resultados indicam que o baixo apoio social, carga elevada de trabalho, alta demanda e baixo controle sobre o trabalho foram os fatores mais investigados e com presença de significância estatística quanto a associação com o adoecimento de professores.

## INTRODUCTION

The International Labor Organization (ILO)^1^ defines psychosocial factors at work as those referring to work environment interactions, job content, organizational factors and skills, and individual features of labor that may have a negative influence on individuals’ health and satisfaction with their jobs. It is worth highlighting that, in order to better understand that concept, we must also consider individual labor perceptions and experiences influenced by the socioeconomic context.^1^

The teaching task has affective value in the teaching profession.^2^ Environments with a positive organizational climate are more likely to promote the sense of well-being. However, professional satisfaction can be influenced by different job-routine factors, such as participation in decision-making, autonomy, social support, and remuneration, among others.^3^

Epidemiological data about teachers’ illness and absence from work due to health issues have shown that morbidities often observed in this population are linked to mental, physical, and vocal health.^4,5^ Psychological demands, such as low control and lack of social support, cause distress and are likely to damage workers’ health; moreover, they can embody different meanings for labor force groups within their cultural, social, and occupational contexts. The literature has also demonstrated that social support has a positive influence on the health of individuals and their satisfaction with their jobs.^6^

Given the importance of including psychosocial factors in the list of triggers and aggravating factors for the development of diseases, it is necessary to better understand those that negatively influence the health of teachers. With that approach, this literature review may contribute to scientific knowledge by assessing the psychosocial aspects of labor that trigger teachers’ illness.

Consequently, results in the current review may help guide further improvements in labor conditions and the elaboration of practices focusing on teachers, in order to prevent losses in these workers’ health. Accordingly, the aim of the present study is to analyze scientific evidence about associations between psychosocial factors at work and teachers’ illness.

## METHODS

A systematic literature review of articles on psychosocial factors at work and teachers’ illness published in the last 11 years (2011-2021) was conducted. Study protocol was recorded at the International Prospective Register of Systematic Reviews (PROSPERO), under number CRD42021234983. Preferred Reporting Items for Systematic Reviews and Meta-Analyses (PRISMA)^7^ recommendations were followed and used to build this review.

### ELIGIBILITY CRITERIA

Criteria used for article selection were I - to be published in Portuguese, English, or Spanish; II - to have investigated psychosocial factors at work; III - to have included teachers who act at any teaching level, except for higher education, as assessed population; IV - to have assessed the presence of morbidities in teachers; and V - to be available for full-text access.

Qualitative methodology studies, article reviews, editorials, opinions, comments, dissertations and theses in repositories, and articles with methodological quality classified as “weak” by the Quality Assessment Tool for Quantitative Studies (QATQS)^8^ were excluded from the review.

### BIBLIOGRAPHICAL SEARCH STRATEGY

The following electronic databases were consulted: Biblioteca Virtual em Saúde (BVS), Medical Literature Analysis and Retrievel System Online (Medline), Cumulative Index of Nursing and Allied Health Literature (Cinahl), Scopus, Web of Science (WoS), PsycINFO, and Excerpta Medica Database (Embase). The search strategy combined selected descriptors (Descritores em Ciências da Saúde/Medical Subject Headings [DeCS/MeSH]) that are described in Chart 1. Electronic searches started in February 2021 and finished in April 2021.

**Chart 1:** Search strategy records: bases, descriptors, and Boolean operators

Base	Strategy
BVS Portal^*^	(docentes OR faculty OR docentes OR “corps enseignant” OR “corpo docente” OR docente OR educador OR educadores OR professor OR professores OR teacher OR teachers) AND (“doenças profissionais” OR “occupational diseases” OR “enfermedades profesionales” OR “maladies professionnelles” OR “doenças ocupacionais” OR “doenças do trabalho” OR “saúde do trabalhador” OR “occupational health” OR “salud laboral”) AND (“impacto psicossocial” OR “psychosocial impact” OR “impacto psicosocial” OR “impact psychosocial” OR “apoio social” OR “social support” OR “apoyo social” OR “soutien social” OR “fatores psicossociais” OR “psychosocial factors”) AND ( db:(“LILACS” OR “IBECS” OR “BDENF” OR “INDEXPSI”))
MEDLINE (via PubMed)	(faculty OR professor OR teacher OR teachers) AND (“occupational diseases” OR “occupational health”) AND (“psychosocial impact” OR “social support” OR “psychosocial factors”)
CINAHL SCOPUS WoS PsycINFO (via Capes Portal)	(faculty OR professor OR teacher OR teachers) AND (“occupational diseases” OR “occupational health”) AND (“psychosocial impact” OR “social support” OR “psychosocial factors”)
EMBASE^*^ (via Capes Portal)	(professor OR teacher) AND (‘occupational diseases’ OR ‘occupational health’) AND (‘psychosocial factors’)

* MEDLINE results were excluded from the following databases: BVS and EMBASE.

BVS = Biblioteca Virtual em Saúde; CAPES = Coordenação de Aperfeiçoamento de Pessoal de Nível Superior; CINAHL = Cumulative Index of Nursing and Allied Health Literature; Embase = Excerpta Medica Database; Medline = Medical Literature Analysis and Retrievel System Online; WoS = WEB OF SCIENCE.

### STUDY SELECTION

After removing duplicate publications in the first stage, initial screening consisted of reading all titles and abstracts in order to select the ones meeting the eligibility criteria. Subsequently, selected articles were fully read for extraction of the following data: authors, publication year, country where the study was conducted, sample features, investigated psychosocial factors, instrument for psychosocial factors’ evaluation, main findings, health outcome, and methodological quality assessment.

### STUDY QUALITY ASSESSMENT

Collected data were recorded and organized in an Excel database. All selection processes in this review were conducted by independent peers. Disagreements were solved by consensus and, whenever reaching a consensus was not possible, the case was assessed by a third researcher.

The methodological quality of the selected articles was individually and independently analyzed by two raters. The QATQS tool, which was developed by the Effective Public Health Practice Project (EPHPP) research group,^8^ was used in the analysis.

QATQS presents 22 items that are divided into 8 blocks, labeled from A to H. The following items are assessed: A - selection bias; B - study design; C - confounders; D - blinding; E - data collection method; F - withdrawals and dropouts; G - intervention integrity; and H - analyses. Each block can be classified as 1 - strong (no weak ratings), 2 - moderate (one weak rating), or 3 - weak (two or more weak ratings). A global index that classifies the study based on one of the categories is found by the end of the evaluation process.

## RESULTS

**Figure 1:**
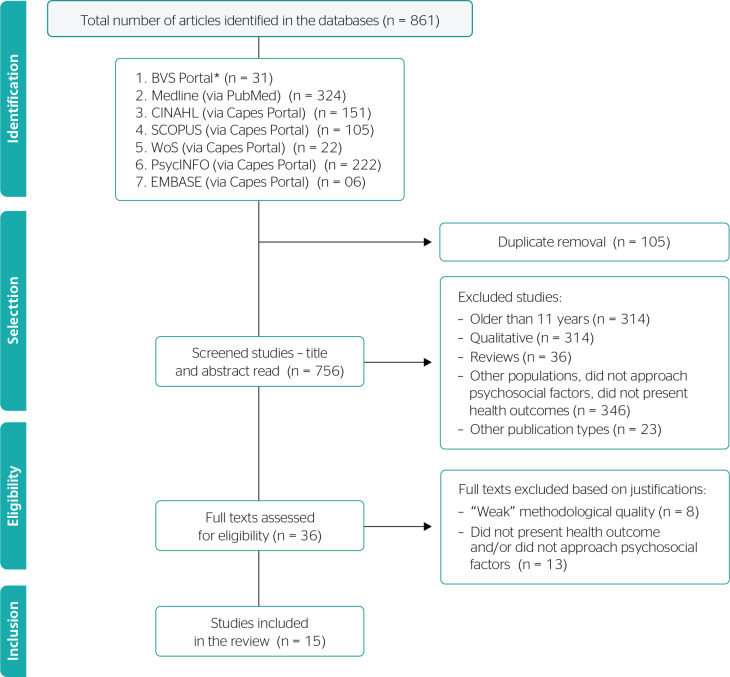
Flowchart describing the study selection process. Created by the authors, adapted from Preferred Reporting Items for Systematic Review and Meta-Analyses (PRISMA). * MEDLINE results were excluded from the following databases: BVS and EMBASE. BVS = Biblioteca Virtual em Saúde; CAPES = Coordenação de Aperfeiçoamento de Pessoal de Nível Superior; CINAHL = Cumulative Index of Nursing and Allied Health Literature; Embase = Excerpta Medica Database; Medline = Medical Literature Analysis and Retrievel System Online; WoS = WEB OF SCIENCE.

The search strategy led to 861 studies in the investigated databases; after the eligibility criteria were applied, only 15 articles remained for the review. Figure 1 shows the flowchart of the article selection process. Studies in the current review are distributed through the European (n = 5),^9-13^ Asian (n = 3),^14-16^ African (n = 2),^17,18^ Oceania (n = 1),^19^ North American (n = 1),^20^ and South American continents (n = 3).^21-23^

All eligible articles were cross-sectional, and most of them had mostly female teachers in their samples. The included manuscripts were published between 2011 and 2021, and English was the prevailing publication language. According to evaluation criteria in the QATQS/EPHPP^8^ instrument, the quality of almost all articles was classified as “moderate” (n = 15).^9-23^

Among teachers’ illness causes, the studies reported common mental disorders (CMD) - anxiety, depression, and emotional distress (53.3%, n = 8),^9,10,12-14,19-21^ musculoskeletal pain (MSP) - pain in the low back, shoulders, arms, legs (26.7%, n = 4),^15,17,18,23^ and burnout syndrome (6.6%, n = 1).^22^ Another 2 studies identified the combination of depression and burnout syndrome (6.6%, n = 1)^11^ and depression and MSP (6.6%, n = 1).^16^

Psychosocial factors associated with teachers’ illness included low social support,^12,13,16-18,21^ low job control,^12,16,17,20,23^ heavy workload,^9,11,14,15,21,22^ high job demands,^13,17,23^ organizational climate,^11,19^ role ambiguity,^14,21^ effort/award unbalance,^11^ low family support,^14^ teacher-student relationship,^10^ and intimidation at the workplace.^12^

Different instruments were used to assess psychosocial aspects. The Job Content Questionnaire (JCQ) was adopted in five studies (33.3%),^12-14,17,23^ and 2 studies used the Unidad de Investigación Psicosocial de la Conducta Organizacional (UNIPSICO) (13.3%).^21,22^ Other validated instruments were also cited in the articles (26.7%, n = 4),^9,11,16,19^ as shown in Table 1. The remaining studies (26.7%, n = 4)^10,15,18,20^ used non-validated instruments, such as questionnaires exclusively developed for a given research study and information collected in secondary databases.

**Table 1:** General features of articles published between 2011 and 2021 about psychosocial factors and teachers’ illness

	Authors, year, and study country	Sample (n/%)	Psychosocial factors	Evaluation instruments	Main findings	Health outcome
1	Cardoso et al. (2011/Brazil)^23^	n = 3,197 91.4% women 8.6% men	Demand-control	JCQ - Brazilian version Validated	High MSP prevalence in teachers working with high demand practices and active work.	MSP (lower limbs, upper limbs, and back)
2	Borrelli et al. (2014/Italy)^13^	n = 113 90% women 10% men	Job demand; social support	JCQ - Italian version Validated	Low mental health levels in teachers were statistically associated with high job demands and with low social support.	CMD
3	Erick & Smith (2014/Botswana)^17^	n = 1,747 72.7% women 27.3% men	Demand-control; social support at work	JCQ - English version Validated	The investigated psychosocial factors did not present statistical significance for low back pain.	Low back pain
4	Garrick et al. (2014/Australia)^19^	n = 960 75% women 25% man	Organizational climate at work	PSC-12 Validated	Teachers working in schools with the highest elationship and psychosocial safety indices showed the lowest levels of psychological disorders.	CMD
5	Baka et al. (2015/Poland)^11^	n = 316 79% women 21% men	Interpersonal conflict; organizational restrictions; workload	ICAWS Validated	High organizational restrictions were closely related to depression. High job demands were closely related to high job burnout.	Depression and burnout syndrome
6	Carlotto & Câmara (2015/Brazil)^21^	n = 679 91.8% women 8.1% men	Social support; ambiguity and role conflict; work overload	UNIPSICO Validated	Teachers who observed ambiguity in their role at work, excessive workload, low social support, and low self-effectiveness were more prone to having mental disorders.	CMD
7	Hinz et al. (2016/Germany)^9^	n = 1,024 86.2% women 13.8% men	Effort and reward; workload	ERI Validated	Teachers working full-time shifts reported the highest effort-reward indices. Full-time jobs were closely related to mental health issues.	CMD
8	Nakada et al. (2016/Japan)^14^	n = 1,006 59.6% women 40.4% men	Workload; conflict and role ambiguity; social support from family and friends	GJSQ - Japanese version Validated	Heavy workload, conflict and role ambiguity at work, and low social support from family and friends were closely associated with depression symptoms.	Depression symptoms
9	Ehsani et al. (2018/Iran)^15^	n = 586 66% women 44% men	Workload	Questionnaire developed for the research Not validated	Heavy workloads related to working in computers and doing test corrections were closely related to neck pain.	Neck pain
10	Harding et al. (2018/England)^10^	n = 1,182 63.6% women 36.4% men	Social elationship at work (teacher/student relationship)	Quality evaluation of teacher-student relationships consisted of one single question Not validated	There was a statistical association between good teacher-student relationships and decreased depression symptoms in teachers.	Depression symptoms
11	Carlotto & Câmara (2019/Brazil)^22^	n = 679 91.8% women 8.1% men	Autonomy; role conflict; role ambiguity; work overload; social support; interpersonal conflict; negative feedback	UNIPSICO Validated	Work overload and low social support were closely associated with burnout events.	Burnout syndrome
12	Elias et al. (2019/Kenya)^18^	n = 417 60.6% women 39.4% men	Social support	Questionnaire developed for the research Not validated	Low social support from supervisors was closely associated with MSP.	MSP
13	Jones-Rincon & Howard (2019/USA)^20^	n = 3,003 86.3% women 13.7% men	Job control	Instrument adapted from the US Education Department, NCES (2010) Not validated	Teachers with anxiety disorder reported having less control over their work.	Anxiety
14	Malinauskiene et al. (2019/Lithuania)^12^	n = 517 81.1% women 18.9% men	Demand-control; social support; intimidation at work	JCQ - Swedish version Validated	Lower job control, low social support, and intimidation at work were closely related to psychological disorders.	CMD
15	Ng et al. (2019/Malaysia)^16^	n = 367 86.6% women 13.4% men	Job control	WOAQ Validated	Low job control and lower social support from workmates were closely related to MSP.	Depression and MSP

CMD = common mental disorder; ERI = effort-reward imbalance model; GJSQ = Generic Job Stress Questionnaire; ICAWS = Interpersonal Conflicts at Work Scale; JCQ = Job Content Questionnaire; MSP = musculoskeletal pain; NCES = National Center of Education Statistics; PSC-12 = Psychosocial safety climate; UNIPSICO = Unidad de Investigación Psicosocial de la Conducta Organizacional (Battery of Psychosocial Risk Assessment); WOAQ = Work Organization Assessment Questionnaire.

Only one publication (6.6%)^17^ did not show significant statistical associations of demand-control factors and social support with teachers’ illness. Overall methodological quality features of studies and publications included in the present review are described in Table 1.

## DISCUSSION

Scientific evidence suggests an association between psychosocial factors at work and teachers’ illness. Studies showing the presence of mental and physical illness among teachers who reported low social support, heavy workload, high job demand, and lower job control prevailed in the sample. The largest number of publications was observed in 2019,^12,16,18,20,22^ and Brazil was the country accounting for the largest number of studies.^21-23^

Results from different countries reinforce the relevance of acknowledging psychosocial factors related to the workplace when analyzing teachers’ health. Different definitions for psychosocial factors were used, as well as a large number of data collection instruments (most of them were validated). It is worth highlighting that using validated instruments provides more consistent results about the object being measured and opens room for comparisons between results from several studies.

Based on the present review, low social support, heavy workload, high job demand, and low job control are associated with the presence of mental disorders,^9,13,16,19-21^ MSP,^15,17,18,23^ and burnout syndrome.^11,22^ Social support is indicative of social environment quality at work, of the relationship between employees and managers, and of the relationship among workmates.^24^ According to Araújo & Karasek,^6^ this aspect is now assessed within the demand-control model, based on the model proposed by Johnson & Hall.^25^ It has been identified as an important mediator between demand and control effects and impacts on workers’ health.

Accordingly, our findings reinforce such data by showing that not receiving social support is associated with MSP, depression symptoms, and professional exhaustion. However, it is important highlighting that teachers who have support at work have such deleterious effects minimized, given that this increases the sense of well-being at the work environment.^26-28^

This result is pertinent to the discussion as it reveals the importance of this support in the teaching profession, whether from colleagues or from managers and workmates. Thus, this finding allows us to direct and suggest practices that can contribute to dealing with the lack of time to update, prepare classes, correct activities, and discuss planning with peers, in addition to the administrative demands of the school, social situations regarding the students, and precarious working conditions.

Frutuoso & Cruz^29^ associated workload with a permanent tension between job demands and workers’ physical and psychological ability to fulfil them. It is clear that the intense work linked to teaching is not limited to the number of hours working at the school but to other labor features that teachers must perform out of the classroom,^30^ resulting in physical and mental exhaustion due to work factors.^31^

Overtime, changes in the teaching process due to educational reforms have caused important transformations in teachers’ actions.^32^ Demands from social context, productivity due to pedagogical and managerial demands, and responsibility for the students’ and schools’ performance are some of the factors influencing work overload.

High job demands perceived by teachers are closely related to low levels of mental health^13^ and high prevalence of MSP.^23^ Social and professional relationships along with responsibilities and commitments beyond the organizational aspects of the job are determining elements in the health conditions of teachers. When work permeates the school environment and functions, schedules are disregarded, and thus daily life becomes more exhausting.

These findings make us rethink the importance of balancing activities based on teachers’ workload in order to make sure that they have enough time to carry them out. It is necessary to rationalize work/rest time, eliminate excessive out-of-class workloads, and promote the updating of academic training and the use of new technologies. Thus, the accumulation of tasks that lead to CMDs and other illnesses experienced by teachers, such as depression and anxiety, can be avoided.

Control over work is related to workers’ ability to be autonomous at decision-making about their own activities. The literature^33^ points to an association between job demands and job control, revealing that individuals exposed to high demands and low control had greater emotional exhaustion and more complaints related to dissatisfaction with work.

The present review, supported by Jones-Rincon & Howard^20^ and Ng et al.,^16^ shows that low control is closely related to anxiety disorders and MSP in teachers. It can be assumed that teachers’ overwork may lead them to dedicate additional efforts to some tasks that appear to be coherent with the time available to perform them, reducing control over their own work with consequent physical and psychological symptoms in the face of this conflict.

Although they are still poorly discussed in the literature, psychosocial factors such as organizational climate,^11,19^ effort reward,^9^ family support,^14^ teacher-student relationship,^11^ and intimidation at work^12^ were found to be associated with teachers’ illness in this review. Many of these professionals also reported a lack of recognition for their work and working in environments where they are even physically threatened. That loss of the sense of work, which has an impact on professional devaluation and dropout,^34,35^ is among the consequences of the psychological assault experienced by teachers.^36^

Some limitations of the present review should be highlighted. For example, there was a lack of studies with high methodological quality, based on QATQS assessment.

Although the cross-sectional design does not allow us inferring a cause-effect relationship between the assessed variables, the present findings enable observing and rethinking the importance of knowing the factors contributing to illness in this group for health surveillance purposes.

The evaluations were conducted by two researchers, who were blinded and independent, in an attempt at minimizing all selection and classification biases in the current review. Although the researchers used combinations and keywords for the bibliographic search, articles related to the topic of interest may not have been reached by the adopted search strategy. One example regards work-related voice disorders, which are some of the most prevalent issues among teachers but were not addressed in any of the selected articles.^37^

Given the evidence provided herein, we suggest that additional studies are performed, based on different research designs and focusing on identifying psychosocial factors and those affecting teachers’ health conditions. This will allow proposing measures and actions related to work organization, social support (both from managers and workmates), as well as making improvements in teachers’ relationships with students and their parents in a more effective and broader way.

## CONCLUSIONS

The investigation about the psychosocial aspects of labor and teachers’ health has been amplified in recent years. Mental illness, MSP, and burnout syndrome are associated with low social support, heavy workload, high job demands, low job control, organizational climate, role ambiguities, low family support, teacher-parent-student relationships, intimidation, and safety at work.

## References

[1] International Labour Organisation (ILO) (1984). Psychosocial factors at work: recognition and control. Report of the
Joint ILO/WHO Committee on Occupational Health.

[2] Ronit B, Nir AE. (2012). The importance of teachers’ perceived organizational support to
job satisfaction What’s empowerment got to do with it?. J Educ Adm.

[3] Alves MG, Azevedo NR, Gonçalves TNR. (2014). Satisfação e situação profissional:
um estudo com professores nos primeiros anos de carreira. Educ Pesqui.

[4] Araújo TMD, Carvalho FM. (2007). Condições de trabalho docente e saúde na
Bahia: estudos epidemiológicos. Educ Soc.

[5] Medeiros AM, Vieira MT. (2019). Work absenteeism due to voice disorders in Brazilian
schoolteachers. Cad Saude Publica.

[6] Araújo TM, Karasek R. (2008). Validity and reliability of the job content questionnaire in
formal and informal jobs in Brazil. Scand J Work Environ Health.

[7] Page MJ, McKenzie JE, Bossuyt PM, Boutron I, Hoffmann TC, Mulrow CD (2021). The PRISMA 2020 statement: an updated guideline for reporting
systematic reviews. BMJ.

[8] Effective Public Healthcare Panacea Project Quality assessment tool for quantitative studies.

[9] Hinz A, Zenger M, Brähler E, Spitzer S, Scheuch K, Seibt R. (2016). Effort-reward imbalance and mental health problems in 1074 German
teachers, compared with those in the general population. Stress Health.

[10] Harding S, Morris R, Gunnell D, Ford T, Hollingworth W, Tilling K (2019). Is teachers’ mental health and wellbeing associated with
students’ mental health and wellbeing?. J Affect Disord.

[11] Baka Ł. (2015). Does job burnout mediate negative effects of job demands on
mental and physical health in a group of teachers? Testing the energetic
process of Job Demands-Resources model. Int J Occup Med Environ Health.

[12] Malinauskiene V, Malinauskas R, Malinauskas M. (2019). Leisure-time physical inactivity and psychological distress in
female-dominated occupations in Lithuania. Women Health.

[13] Borrelli I, Benevene P, Fiorilli C, D’Amelio F, Pozzi G. (2014). Working conditions and mental health in teachers: A preliminary
study. Occup Med.

[14] Nakada A, Iwasaki S, Kanchika M, Nakao T, Deguchi Y, Konishi A (2016). Relationship between depressive symptoms and perceived individual
level occupational stress among Japanese schoolteachers. Ind Health.

[15] Ehsani F, Mohseni-Bandpei MA, Fernández-De-Las-Peñas C, Javanshir K. (2018). Neck pain in Iranian school teachers: Prevalence and risk
factors. J Bodyw Mov Ther.

[16] Ng YM, Voo P, Maakip I. (2019). Psychosocial factors, depression, and musculoskeletal disorders
among teachers. BMC Public Health.

[17] Erick PN, Smith DR. (2014). Low back pain among school teachers in Botswana, prevalence and
risk factors. BMC Musculoskelet Disord.

[18] Elias HE, Downing R, Mwangi A. (2019). Low back pain among primary school teachers in Rural Kenya:
Prevalence and contributing factors. African J Prim Heal Care Fam Med.

[19] Garrick A, Winwood PC, Mak AS, Cathcart S, Bakker AB, Lushington K. (2014). Prevalence and organisational factors of psychological injury
among Australian school teachers. Australas J Organ Psychol.

[20] Jones-Rincon A, Howard KJ. (2019). Anxiety in the workplace: A comprehensive occupational health
evaluation of anxiety disorder in public school teachers. J Appl Biobehav Res.

[21] Carlotto MS, Câmara SG. (2015). Prevalence and risk factors of common mental disorders among
teachers. Rev Psicol Trab Org.

[22] Carlotto MS, Câmara SG. (2019). Prevalence and predictors of burnout syndrome among public
elementary school teachers. Anal Psicol.

[23] Cardoso JP, Araújo TM, Carvalho FM, Oliveira NF, Reis EJFB. (2011). Aspectos psicossociais do trabalho e dor
musculoesquelética em professores. Cad Saude Publica.

[24] Moreira DZ, Rodrigues MB. (2018). Saúde mental e trabalho docente. Estud Psicol.

[25] Johnson JV, Hall EM. (1988). Job strain, work place social support, and cardiovascular
disease: a cross-sectional study of a random sample of the Swedish working
population. Am J Public Health.

[26] Birolim MM, Mesas AE, González AD, Santos HG, Haddad MCFL, Andrade SM. (2019). Trabalho de alta exigência entre professores:
associações com fatores ocupacionais conforme o apoio
social. Cienc Saude Colet.

[27] Camada IM, Araujo TM, Porto LA. (2016). Trabalho docente e saúde mental: a importância do
apoio social. Cad Educ.

[28] Glina DMR, Rocha LE. (2010). Saúde mental no trabalho: da teoria à
prática. Rev Bras Saude Ocup.

[29] Frutuoso JT, Cruz RM. (2005). Mensuração da carga de trabalho e sua
relação com a saúde do trabalhador. Rev Bras Med Trab.

[30] Garcia MMA, Anadon SB. (2009). Reforma educacional, intensificação e
autointensificação do trabalho docente. Educ Soc.

[31] Gasparini SM, Barreto SM, Assunção AA. (2006). Prevalência de transtornos mentais comuns em professores
da rede municipal de Belo Horizonte, Minas Gerais, Brasil. Cad Saude Publica.

[32] Brasil, Presidência da República, Casa Civil,
Subchefia para Assuntos Jurídicos (1996). Lei nº 9.394/96, de 20 de dezembro de 1996.

[33] De Jonge J, Dollard MF, Dormann C, Le Blanc PM, Houtman ILD. (2000). The Demand-Control Model: specific demands, specific control and
well-defined groups. Int J Stress Manag.

[34] Souza KOJ. (2012). Violência em escolas públicas e a
promoção da saúde: relatos e diálogos com alunos
e professores. Rev Bras Promoç Saude.

[35] Scheibe L. (2020). Valorização e formação dos
professores para a educação básica: questões
desafiadoras para um novo plano nacional de
educação. Educ Soc.

[36] Castro REF, Souza MA. (2012). Efeitos da agressividade infantil para o sofrimento
psíquico de professores em diferentes momentos de
carreira. Estud Psicol.

[37] Masson LM, Ferrite S, Pereira LMA, Ferreira LP, Araújo TM. (2019). Em busca do reconhecimento do distúrbio de voz como
doença relacionada ao trabalho: movimento
histórico-político. Cienc Saude Colet.

